# Case Report: A novel *de novo SPI1* mutation identified in a Chinese patient with agammaglobulinemia

**DOI:** 10.3389/fimmu.2025.1716208

**Published:** 2025-11-28

**Authors:** Qi Peng, Ming Deng, Xiaomei Zeng, Qingqiu Cheng, Mingyu Xie, Siping Li, Xiaomei Lu

**Affiliations:** 1 Laboratory Department, Dongguan Children’s Hospital, Dongguan, Guangdong, China; 2 Department of Medical and Molecular Genetics, Dongguan Institute of Pediatrics, Dongguan, Guangdong, China; 3 Key Laboratory for Children’s Genetics and Infectious Diseases of Dongguan, Dongguan, Guangdong, China; 4 Department of Clinical Laboratory, Qingxi Hospital, Dongguan, Guangdong, China; 5 Rare Disease Clinic, Dongguan Children’s Hospital, Dongguan, Guangdong, China

**Keywords:** PU.1 deficiency, SPI1 mutation, agammaglobulinemia, hematopoietic stem cell transplantation, viral meningoencephalitis

## Abstract

**Background:**

PU.1 deficiency, also known as Autosomal Dominant Agammaglobulinemia-10 (AGM10), is a rare primary immunodeficiency caused by mutations in the *SPI1* gene, leading to B cell deficiency and hypogammaglobulinemia. To date, human cases of *SPI1*-related immunodeficiency have been reported in only a limited number of publications, highlighting the scarcity of clinical data and the importance of further characterization.

**Case description:**

We describe a Chinese patient with recurrent respiratory infections, agammaglobulinemia, and profound B cell lymphopenia. Initial genetic screening using a targeted Primary Immunodeficiency Panel did not identify any related pathogenic variants. Subsequent whole-exome sequencing revealed a novel *de novo* nonsense mutation in the *SPI1* gene(NM_003120.3:c.130G>T, p.Glu44Ter). The patient subsequently underwent hematopoietic stem cell transplantation (HSCT). Immunological recovery progressed favorably, with B-cell reconstitution and normalization of immunoglobulin levels occurring by approximately 10 months post-HSCT. However, the clinical course was complicated by severe viral meningoencephalitis occurring around two months post-HSCT, which presented as recurrent fever. Cerebrospinal fluid analysis confirmed infection with cytomegalovirus (CMV) and torque teno virus (TTV). This infection resulted in progressive neurological deterioration and permanent paralysis.

**Conclusion:**

We report the first Chinese case of PU.1 deficiency caused by a novel *SPI1* mutation. Our finding reinforces the need to include SPI1 in diagnostic panels for agammaglobulinemia. Moreover, the severe viral meningoencephalitis after HSCT, despite immune reconstitution, underscores the critical need for aggressive peri-transplant surveillance.

## 1 Introduction

PU.1 deficiency is a primary immunodeficiency disorder caused by heterozygous pathogenic variants in the *SPI1* gene, which is cataloged in the Online Mendelian Inheritance in Man (OMIM) database as Autosomal Dominant Agammaglobulinemia 10 (AGM10, OMIM#619707) (1). Clinically, affected individuals typically present in early childhood with recurrent bacterial and viral infections—most notably of the sinopulmonary tract—along with a profound reduction or absence of circulating B lymphocytes and hypo- or agammaglobulinemia. Some patients also exhibit deficiencies of conventional dendritic cells (cDCs) and plasmacytoid dendritic cells (pDCs) (1–4).

*SPI1* encodes the transcription factor PU.1, which contains an ETS DNA–binding domain that is essential for early B–cell lineage commitment, as well as for the development of myeloid and dendritic cell lineages (4–8). Acting as a pioneer transcription factor, PU.1 binds to closed chromatin regions and recruits chromatin-remodeling complexes to facilitate chromatin opening, thereby enabling access for non-pioneer transcription factors (9–12). PU.1 (SPI1) haploinsufficiency disrupts early B-cell differentiation by restricting chromatin accessibility at lineage-specific regulatory regions, leading to impaired activation of genes essential for B-cell commitment.

To date, only four published studies, encompassing about 20 patients, have documented the human phenotypic spectrum associated with SPI1 mutations (1–4). The first report identified six unrelated individuals with agammaglobulinemia harboring *SPI1* variants that destabilized the PU.1 protein, impaired its nuclear localization, and reduced chromatin accessibility, resulting in developmental arrest of B cells and loss of dendritic cells (1).

Here, we describe the first Chinese patient with PU.1 deficiency, carrying a *de novo* nonsense variant in SPI1 (c.130G>T, p.Glu44Ter). We provide a detailed account of her clinical trajectory, including a comprehensive timeline, immunologic reconstitution data post-HSCT, and the severe neurological complication she encountered. This case expands the known mutational spectrum of SPI1-related immunodeficiency and underscores the critical challenges in its management.

## 2 Case description

### 2.1 Clinical and immunological features

The proband was a female child born in September 2017 to non-consanguineous parents, neither of whom had a history of agammaglobulinemia. Her younger brother was healthy with normal immunoglobulin levels at the time of her diagnosis.

**Figure 1:**
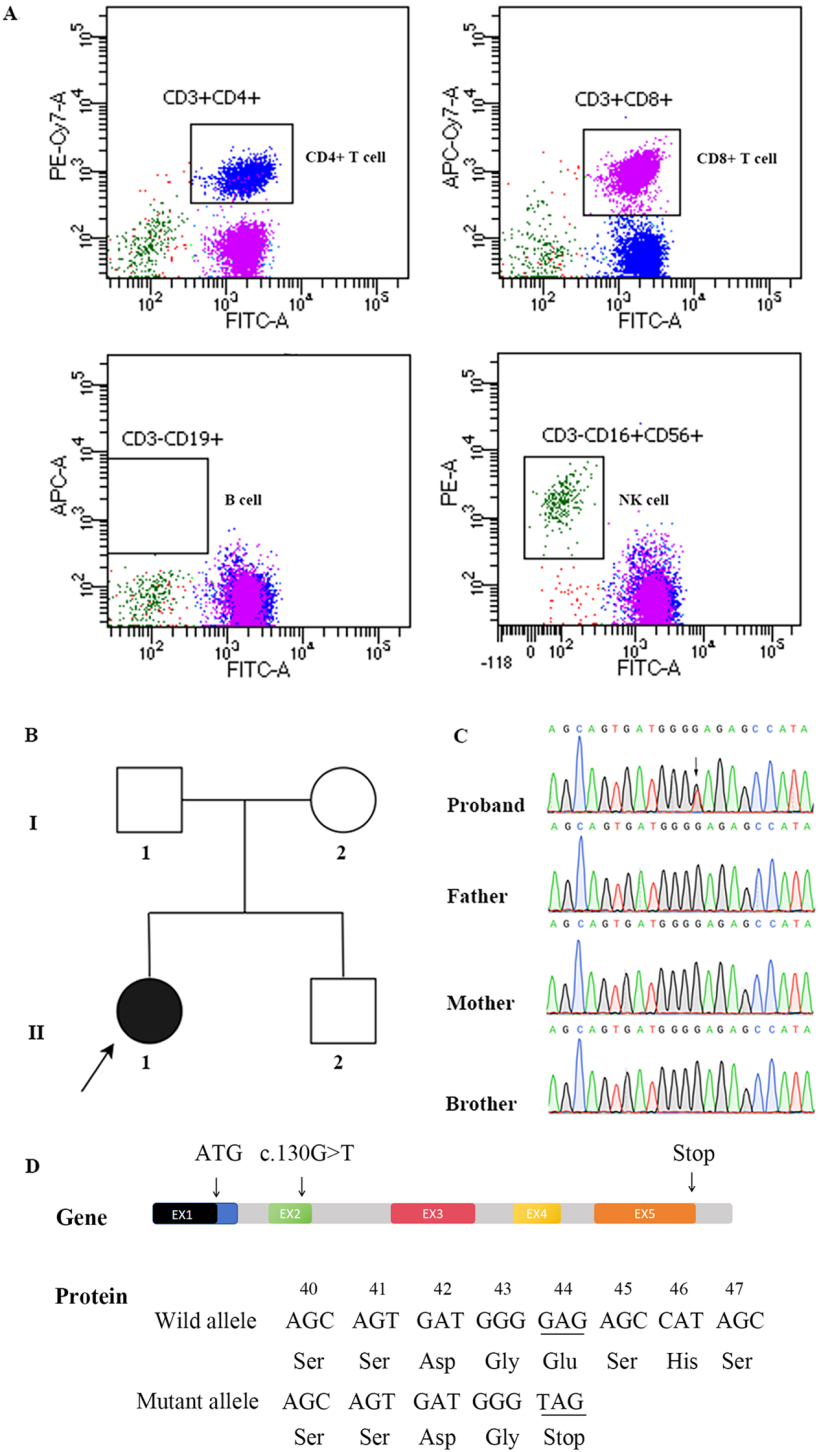
Genetic and immunologic profiling of the proband with AGM10. **(A)** Flow cytometric analysis of peripheral blood lymphocyte subsets from the proband at diagnosis, revealing profound B-cell lymphopenia. **(B)** Pedigree of the family. The arrow indicates the proband with a *de novo* SPI1 variant. **(C)** Sanger sequencing chromatograms of the *SPI1* gene confirming the heterozygous c.130>T (p.Glu44Ter) variant in the proband and its absence in both parents. **(D)** Schematic of the PU.1 protein. The p.Glu44Ter nonsense mutation introduces a premature termination codon (PTC).

At approximately three years of age, the patient began to experience recurrent respiratory tract infections, necessitating multiple hospitalizations at our institution. Laboratory evaluation demonstrated profoundly decreased serum immunoglobulin levels, with IgG at 0.33 g/L (reference range: 3.82–10.58 g/L), IgA at 0.07 g/L (0.14–1.14 g/L), and IgM at 0.04 g/L (0.40–1.28 g/L), consistent with agammaglobulinemia. Flow cytometric analysis of peripheral blood lymphocyte subsets revealed a profound B-cell lymphopenia, characterized by a near-complete absence of CD19^+^ B cells. These findings were strongly indicative of a congenital primary immunodeficiency disorder (**Figure 1A**). The patient was managed with regular intravenous immunoglobulin (IVIG) replacement therapy following diagnosis. Nevertheless, she continued to experience severe and recurrent respiratory infections.

### 2.2 Genetic analysis

Targeted sequencing using a Primary Immunodeficiency Gene Panel detected no related pathogenic or likely pathogenic variants. Subsequently, whole-exome sequencing (WES) identified a heterozygous nonsense variant in *SPI1* (NM_003120.3): c.130G>T (p.Glu44Ter). Sanger sequencing confirmed the variant as *de novo*, as it was absent in both parents and the healthy sibling (**Figures 1B, C**).

The c.130G>T variant, located in exon 2, introduces a premature termination codon at amino acid position 44. This change is predicted to trigger nonsense-mediated mRNA decay or result in the production of a truncated PU.1 protein lacking its essential functional domains (**Figure 1D**). The variant was not reported in population or disease databases, including dbSNP153, ExAC, gnomAD, or HGMD, and was classified as pathogenic according to ACMG/AMP criteria (PVS1, PS2_Moderate, PM2_Supporting).

### 2.3 Hematopoietic stem cell transplantation and early post-transplant course

Given the genetic diagnosis of PU.1 deficiency and the patient’s history of severe, recurrent infections, hematopoietic stem cell transplantation (HSCT) was performed at a specialized transplantation center as a definitive therapeutic intervention.

Approximately two months after HSCT, the patient developed intermittent fever, and cerebrospinal fluid (CSF) analysis confirmed cytomegalovirus (CMV) infection. Three months post-transplant, she was transferred back to our hospital with a diagnosis of viral encephalitis. On admission, she presented with persistent low-grade fever, increased muscle tone in both ankles, bilateral positive Babinski signs, and right ankle clonus. Metagenomic next-generation sequencing (mNGS) of the CSF detected Torque teno virus (TTV) infection. She was treated with corticosteroid pulse therapy, intravenous immunoglobulin, acyclovir, and supportive antiviral therapy.

After approximately 20 days of hospitalization, throat swab mNGS identified Acinetobacter baumannii infection. Brain magnetic resonance imaging (MRI) demonstrated bilateral subcortical white matter signal abnormalities and cerebral atrophy. Her condition stabilized following appropriate treatment, and she was discharged at the family’s request.

### 2.4 Subsequent clinical course and immunological recovery

**Figure 2:**
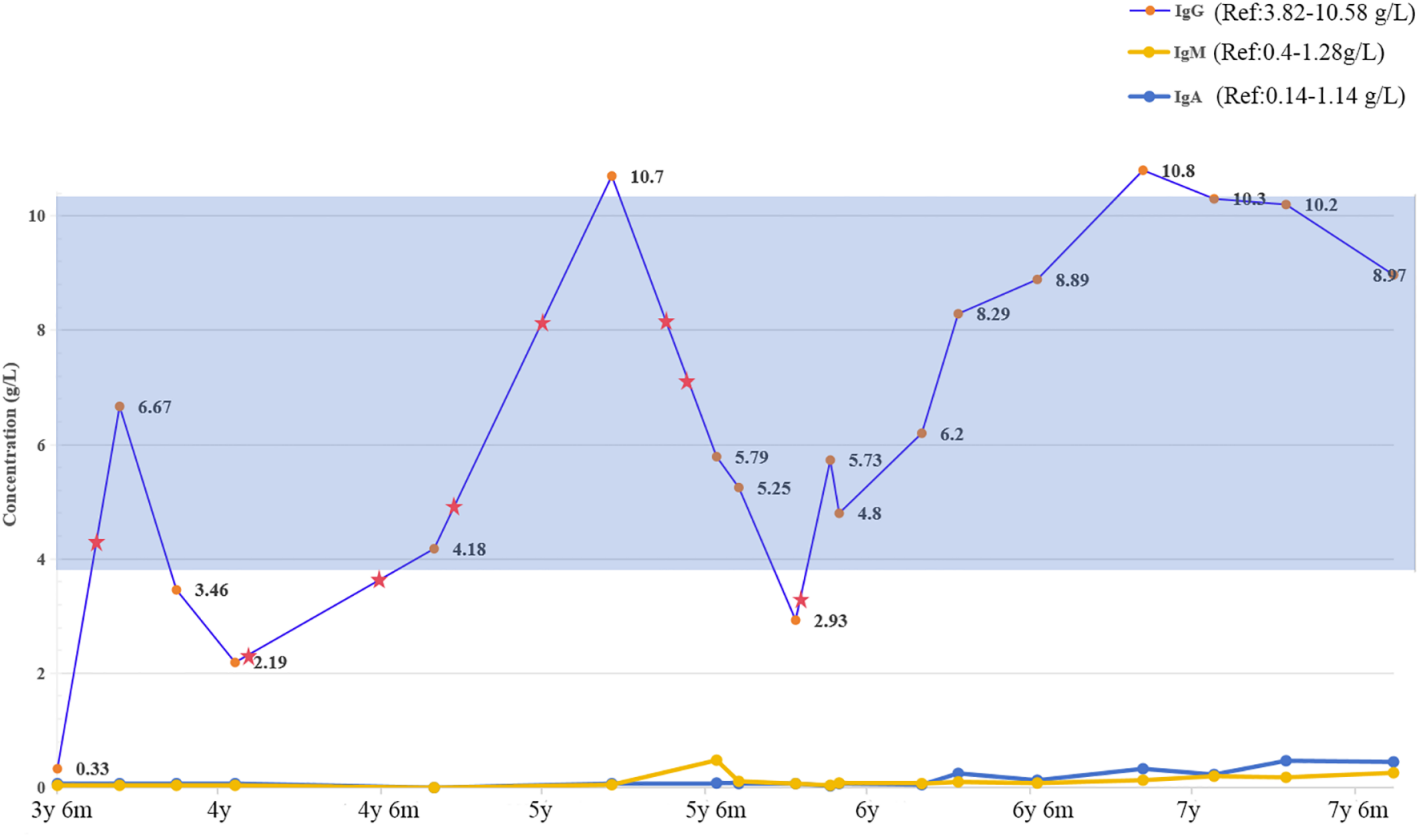
Dynamic changes in serum immunoglobulin levels in the patient over time. The graph illustrates longitudinal measurements of serum IgG, IgA, and IgM concentrations. The blue-shaded area represents the reference range for serum IgG. Pentagram symbols (★) indicate the time points of intravenous immunoglobulin (IVIG) administration.

At approximately five months post-transplant, the patient was re-hospitalized with recurrent fever, chills, and peripheral coldness. Pathogen testing confirmed COVID-19 infection. At this stage, she exhibited marked neuroregression, including loss of motor milestones, inability to sit unsupported, absence of speech, and generalized hypertonia with clenched fists. During the following months, she received regular intravenous immunoglobulin infusions and rehabilitation therapy. By about ten months post-transplant, her B-cell count and immunoglobulin levels had fully normalized, indicating successful immune reconstitution (**Table 1**, **Figure 2**).

**Table 1:** Longitudinal changes in TBNK lymphocyte subsets before and after hematopoietic stem cell transplantation.

Immune parameter	Ref. range (cells/μL)	Pre-Tx (-6M)	Post-Tx +1M	Post-Tx +3M	Post-Tx +10M	Post-Tx +11M
Total Lymphocytes	N/A	2904	2688	1242	6256	5825
T Lymphocytes (CD3^+^)	988-2860	2813	1900	748↓	4222	3731
Helper T Cells (CD3^+^CD4^+^)	650-1440	1278	114↓	70↓	560↓	494↓
Cytotoxic T Cells (CD3^+^CD8^+^)	320-1250	1473	1213	512	3209↑	2828↑
NK Cells (CD3^-^CD56^+^)	150-1000	79↓	774	479	1175↑	1302↑
B Lymphocytes (CD19^+^)	90-560	0↓	0↓	0↓	792↑	633↑

Pre-Tx, pre-transplant; Post-Tx, post-transplant; M, month; Ref. Range, reference range; NK Cells, natural killer cells; N/A, not applicable.

↑ indicates a value above the reference range. ↓ indicates a value below the reference range.

**Figure 3:**
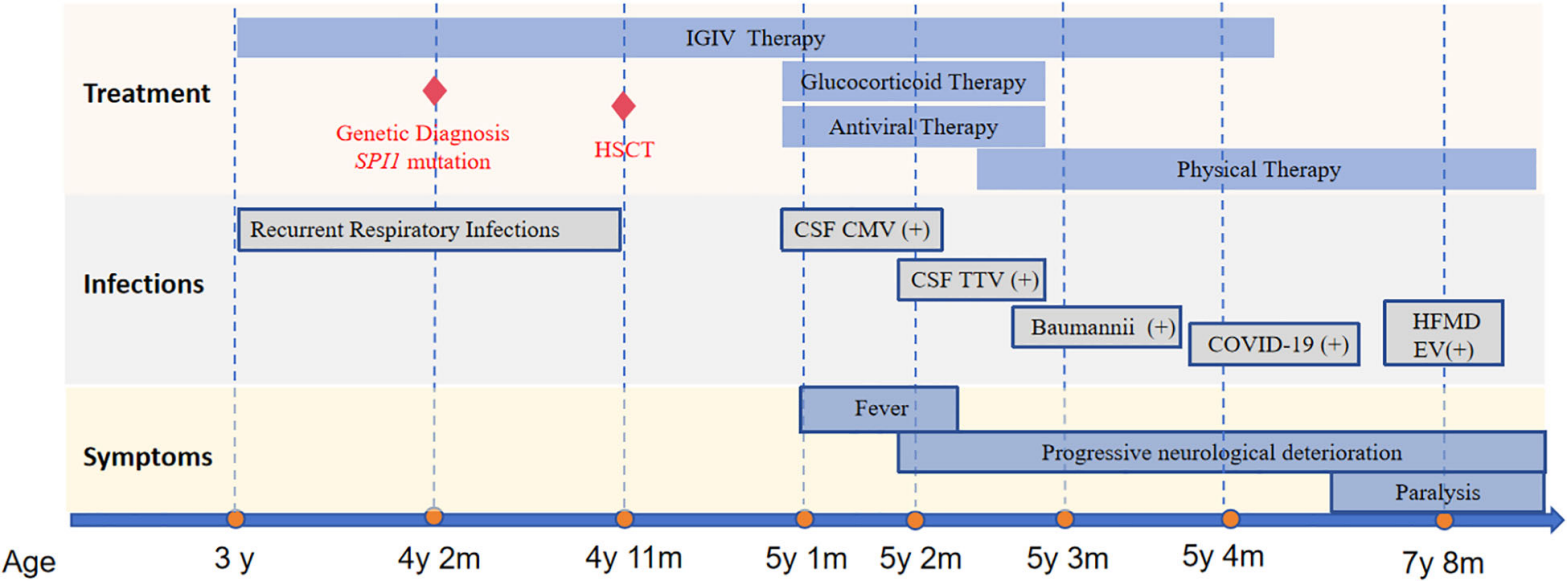
Clinical timeline of the patient with PU.1 deficiency. The blue horizontal bars represent the duration of each clinical event or treatment. The red diamonds indicate key medical milestones, including genetic diagnosis and HSCT.

However, significant neurological sequelae persisted, likely as a consequence of post-encephalitic injury. At the latest follow-up, the patient was alert but had limited visual and auditory tracking, could follow simple one-step commands, and occasionally produced single-syllable vocalizations. Motor function remained severely impaired, with increased muscle tone and poor head control. She could roll to the side but was unable to sit, stand, or walk independently. A detailed clinical timeline is presented in **Figure 3**.

## 3 Discussion

In this study, we describe the first Chinese patient with PU.1 deficiency caused by a heterozygous *de novo* nonsense variant in *SPI1* (c.130>T, p.Glu44Ter). This variant is predicted to introduce a premature termination codon, likely resulting in a truncated and non-functional PU.1 protein due to nonsense-mediated mRNA decay (NMD).This variant is located upstream of the DNA-binding ETS domain of PU.1. Consistent with previous functional studies, such truncating mutations are predicted to impair chromatin accessibility and disrupt B-cell differentiation through haploinsufficiency (1, 4).

To date, only four publications have reported a total of 22 patients with PU.1 deficiency caused by pathogenic *SPI1* variants (1–4). To our knowledge, this is the first documented case of PU.1 deficiency in the Chinese population. Our finding therefore expands both the mutational and ethnic spectrum of this disorder and contributes to the limited global cohort of reported cases. As summarized in **Table 2**, the present case exhibits the characteristic clinical features of PU.1 deficiency, including early-onset infections, profound agammaglobulinemia, and B-cell lymphopenia. The comparison of mutation types, clinical manifestations, and treatment further refines our understanding of the genotypic and phenotypic heterogeneity of PU.1 deficiency. Our case, with its documented profound B-cell lymphopenia, aligns with this core immunologic profile. Infections remain the dominant presentation, furthermore, the spectrum of non-infectious complications is broad, with the enlarged cohort reporting a high incidence of gastrointestinal inflammatory diseases and neurocognitive disorders (1–4), echoing the rapidly progressive neurocognitive decline described previously (2).

**Table 2:** Demographic, genetic, and clinical characteristics of patients with SPI1 mutations.

Pt. ID	Sex	Age at report	Nucleotide change (NM_003120.3)	Protein change (NP_003111.2)	Variant type	Inheritance	Infections	Immunotherapies	Reference
P1	M	55 y	c.100G>T	p.E34X	Nonsense	Unphased	SPI, SEC	IRT	(4)
P2	M	18 y	c.112delT	p.Y38fs*148	Frameshift	Maternal	SPI, IAVM	IVIG	(4)
P3	F	8 yr	c.130G>T	p.Glu44Ter	Nonsense	*De novo*	SPI; Post-transplant CMV and TTV; EV; COVID-19	IVIG, HSCT	This study
P4	M	3 y	c.159C>G, c.157T>G, c.147-155delTTACTGGGA, c.143insCCCCC	p.D48Afs*82	Frameshift	Unphased	SPI, BM	IRT	(4)
P5	M	5 y	c.322_324delGGCinsAG	p.G108Sfs*78	Frameshift	*De Novo*	SPI, EV	IRT, HSCT	(1, 4)
P6	M	11 y	c.328C>T	p.Q110X	Nonsense	*De Novo*	SPI, BM	IRT	(1, 4)
P7	M	41 y	c.363C>A	p.Y121X	Nonsense	*De Novo*	SPI	IRT	(1, 4)
P8	M	11 y	c.407delG	p.G136Afs*50	Frameshift	Paternal	SPI, POM, POC	IRT	(4)
P9	F	38 y	c.438_439insT	p.D147X	Nonsense	Paternal	SPI, BM	IRT, HSCT	(2)
P10	M	21 y	c.441delC	p.D147Efs*39	Frameshift	Unphased	SPI	IRT	(4)
P11	M	12 y	c.536T>C	p.L179P	Missense	*De Novo*	SPI, BI	IRT	(4)
P12	F	65 y	c.538C>T	p.L180F	Missense	Unphased	SPI; PM	IRT	(3)
P13	M	38 y	c.538C>T	p.L180F	Missense	Maternal	SPI, HIA and HIS	IRT	(3)
P14	M	35 y	c.632A>C	p.H211P	Missense	*De Novo*	SPI, EV	IRT	(1, 4)
P15	M	11 y	c.639G>A	p.W213X	Nonsense	Unphased	SPI	IRT, ADM	(4)
P16	F	19 y	c.667A>G	p.M223V	Missense	*De Novo*	SPI, BI	IRT	(4)
P17	M	3 y	c.676C>T	p.Q226X	Nonsense	Unphased	SPI	IRT	(4)
P18	M	8 y	c.693_694delGC	p.L232Afs*53	Frameshift	Unphased	SPI	IRT	(4)
P19	M	15 y	c.701delA	p.N234Tfs*11	Frameshift	Unphased	SPI	IRT, MTX, HCQ, ETN, TOC, ABA, CAM	(4)
P20	F	21 y	c.722T>G	p.V241G	Missense	Unphased	SPI	IRT, MMF	(4)
P21	M	23 y	c.722T>G	p.V241G	Missense	Paternal	SPI	IRT	(4)
P22	M	28 y	c.739_741delAAG	p.K245del	In-frame Del	Paternal	SPI	IRT	(4)
P23	F	33 y	chr11:(47336320-47776156)×1	NA	Deletion	Unphased	SPI	IRT	(4)

ABA, abatacept; ADM, adalimumab; BM, bacterial meningitis; BI, bronchiectasis; CAM, canakinumab; CMV, cytomegalovirus; EV, enteroviremia; ETN, etanercept; F, female; HIA, *Haemophilus influenzae* arthritis; HIS, *H. influenzae* sepsis; HSCT, hematopoietic stem cell transplantation; IAVM, influenza A virus myocarditis; IRT, immunoglobulin replacement therapy; IVIG, intravenous immunoglobulin; M, male; MMF, mycophenolate mofetil; MTX, methotrexate; PM, pneumococcal meningitis; POC, periorbital cellulitis; POM, pneumococcal osteomyelitis; SEC, *S. typhi* enterocolitis; SPI, sinopulmonary infections; TOC, tocilizumab; TTV, Torquetenovirus.

Variable expressivity and incomplete penetrance are also evident: in a Finnish family carrying the p.Leu180Phe variant, clinical severity ranged from severe infections in the proband to a mild phenotype in the mother and absence of symptoms in an older sister (3), and penetrance in the largest cohort was estimated at 81.8% (4), suggesting that genetic modifiers or environmental factors may influence clinical expression of PU.1 deficiency. Importantly for genetic counseling, despite the link between somatic SPI1 mutations and leukemia, no significant increase in hematologic malignancies was observed in the large cohort of germline variant carriers, suggesting that PU.1 haploinsufficiency may not confer a strong leukemic risk (13–17).

The significant challenges and suboptimal outcomes associated with hematopoietic stem cell transplantation (HSCT) in PU.1 deficiency are clearly illustrated by the collective experience of our patient and previously reported cases (2, 4). In our patient, despite successful B-cell reconstitution following HSCT, severe viral meningitis developed, resulting in permanent paralysis and underscoring the profound vulnerability during immune reconstitution. This complication occurred during the early post-engraftment phase, when compounded immune deficiency resulting from the conditioning regimen and ongoing immunosuppression for graft-versus-host disease (GVHD) prophylaxis created a period of extreme susceptibility to opportunistic infections such as cytomegalovirus (CMV). Previous reports further highlight the complexity of HSCT in this disorder: one patient achieved full donor engraftment and naïve B-cell recovery yet remained dependent on immunoglobulin replacement therapy due to persistently undetectable serum IgA and IgM levels; another experienced graft rejection following transplantation from a healthy sister who was later identified as an asymptomatic carrier of the same familial PU.1 variant (2, 4).

This case, characterized by severe viral infection post-transplantation and unique immune cell dynamics, suggests the possible co-occurrence of immune reconstitution inflammatory syndrome (IRIS). IRIS occurs during immune recovery and manifests as an excessive, dysregulated inflammatory response to latent or pre-existing antigens, which can lead to tissue damage. As detailed in **Table 1**, the patient’s immunomonitoring data revealed that after the onset of viral encephalitis, there was a persistent abnormal increase in CD8^+^ T cells and a significant expansion of NK cells, creating an IRIS-prone imbalance dominated by effector cells with insufficient immunoregulatory function. We therefore hypothesize that newly generated CD8^+^ T cells and NK cells, upon recognizing CMV and TTV antigens in the brain, become intensely activated. In the absence of effective immune regulation, this activation triggers an excessive inflammatory response that results in neuronal “bystander injury.” Consequently, the progressive neurological deterioration is more likely attributable to virus triggered IRIS, which is centered on the overactivation of cytotoxic cells, rather than to direct viral damage. This highlights that in the post-transplant management of conditions such as PU.1 deficiency, emphasis should be placed on evaluating the balance of immune reconstitution and the early recognition and intervention of IRIS.

Together, these cases delineate three major challenges in HSCT for PU.1 deficiency: (i) a high risk of life-threatening infections during immune reconstitution, as tragically evidenced by our case, (ii) the possibility of incomplete humoral immune recovery despite successful engraftment, and (iii) the critical importance of comprehensive genetic screening of related donors to avoid transplantation from asymptomatic carriers. These experiences suggest that HSCT for PU.1 deficiency constitutes a high-risk intervention that may not ensure a functional cure. They also emphasize the necessity for meticulous donor selection, aggressive infection prophylaxis (particularly for herpesviruses like CMV), carefully tailored conditioning regimens, and warrant extreme caution in its clinical application.

Notably, the initial failure of the targeted PID gene panel to detect this *SPI1* variant underscores a key limitation of fixed panels: their inability to keep pace with rapidly evolving gene-disease associations. This case highlights the diagnostic value of whole-exome sequencing (WES) in agammaglobulinemia cases of unknown etiology following negative panel results, while also prompting the critical need for commercial assays to undergo regular updates to incorporate newly validated genes like *SPI1*.

## 4 Conclusion

In summary, we report the first Chinese case of PU.1 deficiency caused by a novel SPI1 nonsense variant, confirming the critical role of PU.1 haploinsufficiency in human agammaglobulinemia. These findings underscore the importance of incorporating *SPI1* into diagnostic gene panels for congenital agammaglobulinemia to improve detection rates. The devastating neurological complication following an otherwise successful HSCT serves as a critical reminder of the precarious immune reconstitution phase in these patients. Given the complex risk–benefit profile of hematopoietic stem cell transplantation (HSCT) in PU.1 deficiency, careful consideration is required when planning curative interventions. Future efforts should focus on expanding case identification, performing functional studies to elucidate PU.1-related pathogenesis, and optimizing management strategies, with a particular emphasis on risk mitigation during the transplant process, to improve outcomes for affected patients.

## Data Availability

The datasets presented in this study can be found in online repositories. The names of the repository/repositories and accession number(s) can be found below: SCV006561095 (ClinVar).
